# Supplementary material for: Development of a computer-aided design software for the quantitative evaluation of aesthetic damage
Source: PLoS One. 2019 Dec 18;14(12):e0226322. doi: 10.1371/journal.pone.0226322 (PMC6919621; doi:10.1371/journal.pone.0226322)
Supplement: S1 Code site — 1. (ZIP) [file pone.0226322.s001.zip › [EESC_jr][Projeto]Relatorio_07_21/reports/templates_zip/gentelella-master/vendors/select2/docs/community.html]

---
layout: default
title: Community - Select2
slug: community
---

# Community

Select2 has an active community that enjoys helping each other out.

# Getting support

Having trouble getting Select2 working on your website? Is it not working
together with another plugin, even though you think it should? Select2 has a
few communities that you can go to for help getting it all working together.

1. Try sending a message to the
   Select2 discussion group
   or searching the archives.
2. Ask in the `#select2` channel on
   `chat.freenode.net` or use the
   web irc client.
3. Raise a question on
   Stack Overflow,
   make sure to include the
   "jquery-select2"
   tag.

# Reporting bugs

Found a problem with Select2? Feel free to open a ticket on the Select2
repository on GitHub, but you should keep a few things in mind:

1. Use the
   GitHub issue search
   to check if your issue has already been reported.
2. Try to isolate your problem as much as possible, so we can easily test if
   the issue has been fixed.
3. Once you are sure the issue is with Select2, and not a third party
   library,
   submit a ticket
   to the repository.

You can find more information on reporting bugs in the
contributing guide,
including tips on what information to include.

# Requesting new features

New feature requests are usually requested by the
Select2 community on GitHub,
and are often fulfilled by
fellow contributors.

1. Use the
   GitHub issue search
   to check if your feature has already been requested.
2. Check if it hasn't already been implemented as a
   third party plugin.
3. Please make sure you are only requesting a single feature, and not a
   collection of smaller features.

You can find more information on requesting new features in the
contributing guide.

# Getting involved

You can find more information on getting involved with Select2 in the
contributing guide.
